# Reclassification of four *Polynucleobacter necessarius* strains as representatives of *Polynucleobacter asymbioticus* comb. nov., *Polynucleobacter duraquae* sp. nov., *Polynucleobacter yangtzensis* sp. nov. and *Polynucleobacter sinensis* sp. nov., and emended description of *Polynucleobacter necessarius*

**DOI:** 10.1099/ijsem.0.001073

**Published:** 2016-08

**Authors:** Martin W. Hahn, Johanna Schmidt, Alexandra Pitt, Sami J. Taipale, Elke Lang

**Affiliations:** ^1^​ Research Institute for Limnology, University of Innsbruck, Mondseestrasse 9, A-5310 Mondsee, Austria; ^2^​ Lammi Biological Station, University of Helsinki, Pääjärventie 320, 16900 Lammi, Finland; ^3^​ Leibniz Institut DSMZ-Deutsche Sammlung von Mikroorganismen und Zellkulturen GmbH, Inhoffenstraße 7B, D-38124 Braunschweig, Germany

**Keywords:** Polynucleobacter, revision, New species, average nucleotide identity, ANI, endosymbiont

## Abstract

Genome comparisons based on average nucleotide identity (ANI) values of four strains currently classified as *Polynucleobacter necessarius* subsp. *asymbioticus* resulted in ANI values of 75.7–78.4 %, suggesting that each of those strains represents a separate species. The species *P*. *necessarius* was proposed by Heckmann and Schmidt in 1987 to accommodate obligate endosymbionts of ciliates affiliated with the genus *Euplotes*. The required revision of this species is, however, hampered by the fact, that this species is based only on a description and lacks a type strain available as pure culture. Furthermore, the ciliate culture *Euplotes aediculatus* ATCC 30859, on which the description of the species was based, is no longer available. We found another *Euplotes aediculatus* culture (Ammermann) sharing the same origin with ATCC 30859 and proved the identity of the endosymbionts contained in the two cultures. A multilocus sequence comparison approach was used to estimate if the four strains currently classified as *Polynucleobacter* *necessarius* subsp. *asymbioticus* share ANI values with the endosymbiont in the Ammermann culture above or below the threshold for species demarcation. A significant correlation (R^2^ 0.98, *P*<0.0001) between multilocus sequence similarity and ANI values of genome-sequenced strains enabled the prediction that it is highly unlikely that these four strains belong to the species *P*. *necessarius*. We propose reclassification of strains QLW-P1DMWA-1^T^ (=DSM 18221^T^=CIP 109841^T^), MWH-MoK4^T^ (=DSM 21495^T^=CIP 110977^T^), MWH-JaK3^T^ (=DSM 21493^T^=CIP 110976^T^) and MWH-HuW1^T^ (=DSM 21492^T^=CIP 110978^T^) as *Polynucleobacter asymbioticus* comb. nov., *Polynucleobacter duraquae* sp. nov., *Polynucleobacter yangtzensis* sp. nov. and *Polynucleobacter sinensis* sp. nov., respectively.

The genus *Polynucleobacter* and the species *Polynucleobacter necessarius* were proposed by K. Heckmann and H. J. Schmidt in 1987 for bacterial endosymbionts of freshwater ciliates affiliated with the genus *Euplotes*. Independent investigations demonstrated that these endosymbionts obligately rely on their host cells (Heckmann & Schmidt, 1987; Vannini* et al.*, 2007) and thus represent obligate endosymbionts. This tight relationship between these bacteria and their hosts made it impossible to establish a pure culture representing the type of *P. necessarius* (Heckmann & Schmidt, 1987; Vannini* et al.*, 2007). Thus, the genus *Polynucleobacter* and its type species *P. necessarius* are two of the few prokaryotic taxa not represented by a type strain. The type of the species *P. necessarius* is represented by a description of the endosymbionts contained in the *Euplotes aediculatus* ‘stock 15’ culture (=E24=ATCC 30859) (Heckmann & Schmidt, 1987). This mixed culture consists of the ciliate, algae serving as food for the ciliate, various free-living bacteria and endosymbionts of the genus *Polynucleobacter*. This culture is for several reasons not suitable for many purposes of comparative taxonomic research. The lack of a pure culture limits determination of new taxa by DNA–DNA re-association experiments (DNA–DNA hybridization) or by chemotaxonomic traits, and phenotypical comparisons are restricted to morphological analyses. Owing to these limitations, the taxonomic classification of strains previously isolated as pure cultures from freshwater habitats (Hahn, 2003) was difficult (Hahn* et al.*, 2009). These strains share 16S rRNA gene sequence similarities ≥99 % with *P. necessarius* endosymbionts but dwell as free-living strains in the water column of freshwater lakes and ponds, thus differing from the endosymbionts profoundly in their lifestyle (Hahn* et al.*, 2009, 2012b; Jezberova* et al.*, 2010; Vannini* et al.*, 2007; Wu & Hahn, 2006). Owing to the lack of pure genomic DNA of the endosymbiotic *P. necessarius*, it was not possible to rigorously test if the free-living and the endosymbiotic strains represent the same species. However, for pragmatic reasons, four free-living strains were preliminarily classified as *P. necessarius* strains due to the high 16S rRNA gene sequence similarity (≥99 %) with endosymbiotic *P. necessarius*, tight phylogenetic clustering with endosymbionts in 16S rRNA gene trees and almost identical G+C values of their genomic DNA (Hahn* et al.*, 2009). Because of the profound differences in lifestyle, separation of endosymbiotic and free-living *P. necessarius* strains into two subspecies, i.e. *P. *necessarius** subsp. *necessarius* and *P. *necessarius** subsp. *asymbioticus*, respectively, was proposed (Hahn* et al.*, 2009). In this previous taxonomic study, four free-living strains representing the genus *Polynucleobacter* were characterized phenotypically and chemotaxonomically and classified as *P. *necessarius** subsp. *asymbioticus* with strain QLW-P1DMA-1^T ^as the type strain.

## Evaluation of the taxonomic position of strains previously classified as *P. necessarius *subsp.* asymbioticus*

Recently, genomic and ecological traits of two free-living strains previously described as *P. necessarius* subsp. *asymbioticus* (Hahn* et al.*, 2009) were compared (Hahn* et al.*, 2016). This investigation revealed an average nucleotide identity (ANI) value of 75.6 % for complete genome sequences of strains QLW-P1DMWA-1^T^ and MWH-MoK4^T^, which is far below the species demarcation threshold of 95–96 % ANI suggested for prokaryotic species (Kim *et al.*, 2014; Konstantinidis & Tiedje, 2005a; Konstantinidis *et al.*, 2006).

In the study presented here, we evaluated the taxonomic position of all four free-living strains of the genus *Polynucleobacter* previously classified as *P. necessarius* subsp. *asymbioticus* (Hahn *et al.*, 2009) by genome comparison. We determined the genome sequence of the remaining two strains (MWH-HuW1^T^ and MWH-JaK3^T^) and completed the phenotypic characterization of strain MWH-MoK4^T^.

DNA used for genome sequencing was extracted from biomass grown in liquid NSY medium (Hahn* et al.*, 2004) as described previously for another strain of the genus *Polynucleobacter* (Meincke* et al.*, 2012). Shotgun libraries were paired-end sequenced with an Illumina MiSeq instrument (Eurofins Genomics, Germany). *De novo* assembly of paired-end reads resulted for strains MWH-HuW1^T^ and MWH-JaK3^T^ in 19 and 42 contigs, respectively. In both cases, the genome size is about 2 Mbp (Table 1). Sequencing coverage was about 42× for strain MWH-HuW1^T^ and about 17× for strain MWH-JaK3^T^. The draft genome sequences were annotated using the IMG/ER annotation pipeline (Markowitz* et al.*, 2012) and deposited in DDBJ/EMBL/GenBank (Table 1).

**Table 1.** Major genome characteristics of the six taxa of the genus *Polynucleobacter* compared in this study The upper four strains are currently classified as *P. necessarius* subsp. *asymbioticus* strains (Hahn* et al.*, 2009), the obligate endosymbiont STIR1 is classified as *P. necessarius* subsp. *necessarius* (Boscaro* et al.*, 2013), and strain ‘beta proteobacterium’ CB is lacking a sound classification (Hao* et al.*, 2013) but clusters in 16S rRNA gene (Wang* et al.*, 2009) and other phylogenetic trees (Figs 1 and 2) with *P. necessarius* strains.

Strain	Lifestyle	Genome size (Mbp)	DNA G+C content (mol%)	DDBJ/EMBL/GenBank accession number	IMG Genome ID	Reference
MWH-HuW1^T^ (=DSM 21492^T^)	Free-living	2.32	45.5	LOJJ00000000	2630969031	This study
MWH-JaK3^T^ (=DSM 21493^T^)	Free-living	2.05	45.4	LOJI00000000	2608642177	This study
QLW-P1DMWA-1^T^ (=DSM 18221^T^)	Free-living	2.16	44.8	CP000655	640427129	Meincke *et al.* (2012)
MWH-MoK4^T^ (=DSM 21495^T^)	Free-living	2.03	45.2	CP007501	2505313000	Hahn *et al.* (2016)
'beta proteobacterium' CB	Free-living	2.05	46.1	CP004348	2565956558	Hao *et al.* (2013)
STIR1 (*Euplotes aediculatus*)	Endosymbiont	1.56	45.6	CP001010	2503982034	Boscaro *et al.* (2013)

The four strains previously classified as *P. necessarius* subsp. *asymbioticus*, i.e. strains QLW-P1DMWA-1^T^, MWH-MoK4^T^, MWH-HuW1^T^ and MWH-JaK3^T^, shared >99 % 16S rRNA gene sequence similarity and differed only marginally in genome size and G+C content of their DNA (Table 1). However, they differed in gene content (Table 2). Some of these gene content features were related to previously determined phenotypic traits. This included presence/absence of genes coding for utilization of urea as nitrogen source, catalase genes and genes encoding flagella proteins. In all three cases, gene content data are in partial conflict with previously reported phenotypic features of the strains. Growth on urea as sole nitrogen source was found previously in two strains (Hahn* et al.*, 2009), but genes putatively encoding a urease and urea transporters were only detected in one of the four strains (Table 2). All four strains had been tested previously to be catalase positive, but genes putatively encoding this trait were only detected in two strains. All four strains had been tested by using soft agar plates as being non-motile. But on a closer look, strain MWH-MoK4^T^ colonies may slowly spread on such plates, building swarms up to a diameter of 50 mm within 17 days, and this strain encoded the whole set of genes required for flagella synthesis. Interestingly, a recent microscopical investigation of this strain resulted in observation of a single cell spinning around its length axis. As previously reported, strain MWH-MoK4^T^ also encodes a complete cluster of genes for synthesis of an anoxygenic photosynthesis system but cultures of the strain never showed any pigmentation revealing this trait (Hahn* et al.*, 2016). It has to be considered that some of these differences in gene content and phenotype may simply result from lack of expression of the genes under the cultivation conditions used. In other cases, the discrepancies could result from annotation errors or insufficient phenotypic tests.

**Table 2.** Differences in gene content between the four strains of the genus *Polynucleobacter* investigated taxonomically The table represents an incomplete list of differences between genomes.

Genes putatively encoding:		QLW-P1DMWA-1^T^	MWH-JaK3^T^	MWH-HuW1^T^	MWH-MoK4^T^
Inorganic nutrients					
	ABC-type Fe^3+^ transport system	−	+	+	+
	*feoAB* genes (uptake of Fe^2+^)	+	+	+	−
	ABC-type nitrate/nitrite/cyanate transporter	+	+	−	−
	Nitrate reductase (assimilatory)	+	+	−	−
	Nitrite reductase (assimilatory)	+	+	−	−
	Cyanate lyase (releases NH_3_ and CO_2_ from cyanate)	+	+	−	−
	Urease and ABC-type urease transporter	+	−	−	−
Oxidative phosphorylation/energy metabolism					
	Cytochrome bd-I terminal oxidase (CydAB)	+	−	+	−
	Fumarate reductase	−	+	−	+
	Carbon monoxide dehydrogenase	−	+	−	+
	Acetate permease *actP*	+	−	−	−
Anoxygenic photosynthesis					
	Photosynthesis gene cluster	−	−	−	+
Motility					
	Flagella genes	−	−	−	+
Oxidative stress					
	Catalase	2 genes	1 gene	−	−
Other					
	Cellulose synthase operon protein C	+	−	−	−
	Cellulose synthase catalytic subunit [UDP-forming]	+	−	−	−

For evaluation of the taxonomic position of the four strains, we first tested which strains have to be considered to belong to the same species. Determination of pairwise ANI values by using the software JSpecies (Richter & Rosselló-Móra, 2009) resulted for all combinations in values in the range between 75.7 and 78.4 % (Table 3). Thus, all values are far below the threshold value of 95–96 % ANI suggested for the demarcation of prokaryotic species (Kim* et al.*, 2014; Konstantinidis & Tiedje, 2005; Konstantinidis* et al.*, 2006).

**Table 3.** Comparison of genomic similarity of taxa by pairwise calculation of ANI values

	QLW-P1DMWA-1^T ^	MWH-MoK4^T^	MWH-JaK3^T^	MWH-HuW1^T^
STIR1 (*Euplotes aediculatus*)	78.0	76.1	84.1	76.3
QLW-P1DMWA-1^T^ (=DSM 18221^T^)		75.7	78.3	75.7
MWH-MoK4^T^ (=DSM 21495^T^)			76.4	78.4
MWH-JaK3^T^ (=DSM 21493^T^)				76.7

Next, we aimed to test if at least one of those four free-living strains may belong to the species *P. necessarius* represented by the endosymbionts of the genus *Polynucleobacter* in the mixed *Euplotes aediculatus* ‘stock 15’ culture (ATCC 30859). Unfortunately, ATCC removed this ciliate culture from the catalogue a few years ago. On enquiry, ATCC staff stated, ‘item 30859 has proved too difficult to be able to make a new distribution lot and will likely not ever be available again. From the beginning the item was very difficult to work with and all recent attempts have resulted in complete failure’ (April 2014). These difficulties were responsible for a delivery period of about 10 months for an order of this culture placed previously by one of us in 2003. The culture finally received enabled us to better characterize the type of *P. necessarius* contained in this ciliate culture by resequencing of the 16S rRNA gene and by establishment of 16S–23S intergenic transcribed spacer (ITS) sequences (Vannini* et al.*, 2007). Despite intensive independent efforts in two laboratories, we were not able to maintain the ciliate culture over longer periods of time. Recent searches for other sources of the culture ‘stock 15’ deposited by Heckmann Schmidt at ATCC in 1987 were unsuccessful. The Heckmann culture collection at the University of Munster, Germany, which was mentioned by Heckmann & Schmidt (1987) as a source of the culture, does not exist anymore, and culture requests to this university in June 2015 were unsuccessful.

**Fig. 1.**
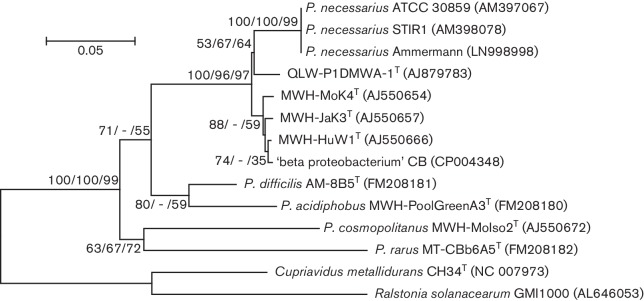
Neighbour-joining (NJ) tree based on 16S–23S ITS sequences reconstructing the phylogenetic position of endosymbionts representing the genus *Polynucleobacter* contained in the culture of *Euplotes aediculatus* strain Ammermann. Results from analyses by the maximum-likelihood (ML) and maximum-parsimony (MP) methods are also indicated. Bootstrap values (percentage of replicates) above the threshold of ≥60 % are shown for those nodes supported in at least one of the three methods; these bootstrap values are depicted in the order NJ/ML/MP. Bar, 0.05 substitutions per nucleotide position.

The *Euplotes aediculatus* culture investigated by Heckmann & Schmidt (1987) had been isolated by Dieter Ammermann ‘from ponds near Marseille, France' in 1969 (Rao & Ammermann, 1970) and was later provided to Klaus Heckmann. The ciliate bearing the endosymbiotic bacteria was initially identified as* Euplotes eurystomus* (Rao & Ammermann, 1970) but later corrected as *Euplotes aediculatus* (Ammermann, 1971), which is highly similar to the former species. Importantly, the culture of *Euplotes aediculatus* established by D. Ammermann is still maintained at the Muséum National d’Histoire Naturelle (MNHN), Paris, France, and can be obtained from this institution if a material transfer agreement is signed. This culture and the culture ATCC 30859 both descend from the culture established by D. Ammermann in 1969; thus, both should contain the same endosymbionts of the genus *Polynucleobacter*. We obtained the *Euplotes aediculatus* Ammermann culture from MNHN and tested whether the endosymbionts, designated here as *P. necessarius* strain Ammermann, possessed the same 16S–23S ITS sequences as the endosymbionts contained in the previous ATCC culture. Primers used for amplification and sequencing are listed in Table S1 (available in the online Supplementary Material). The 16S–23S ITS sequence of *P. necessarius* strain Ammermann was found to be identical (Fig. 1) to the sequence obtained previously from culture ATCC 30859 (Vannini* et al.*, 2007). blast searches with the ITS sequence obtained revealed that among the 224 ITS sequences of strains representing the genus *Polynucleobacter* currently deposited in DDBJ/EMBL/GenBank, only two organisms share an identical sequence with *P. necessarius* strain Ammermann. These are the endosymbionts contained in ATCC 30859 and *P. necessarius* STIR1 contained in another culture of *Euplotes aediculatus* (Petroni* et al.*, 2002; Vannini* et al.*, 2007). All other ITS sequences of strains representing the genus *Polynucleobacter* share similarities in the range of 77–97 %. This suggests that the endosymbionts in the culture obtained from the MNHN are indeed identical with the type material of *P. necessarius* contained in ATCC 30859.

The most straightforward strategy for comparison of the four free-living strains with the endosymbionts would have been genome sequencing of the endosymbionts contained in the *Euplotes aediculatus* Ammermann culture. However, this would be a non-routine task because the endosymbionts comprise only a very small fraction of the total DNA contained in the culture, and mass cultivation for yielding DNA amounts sufficient for sequencing of the endosymbiont genome with an acceptable coverage would be quite laborious. Instead of whole genome sequencing, we employed a multilocus sequencing approach with subsequent sequence comparisons and phylogenetic analyses. Assuming that *P. necessarius* strain Ammermann shares a high genome similarity with the completely sequenced *P. necessarius* STIR1 (Boscaro* et al.*, 2013), we selected eight loci representing housekeeping genes scattered around the STIR1 genome and designed specific primers for partial amplification (Table S1). These primers enabled sequencing of the eight loci of *P. necessarius* strain Ammermann resulting in a total sequence length of 6087 bp. The concatenated sequence of the endosymbiont and the sequences extracted from the genomes of the four free-living strains possessed sequence similarities in the range of 81.8–88.4 % (1108–704 nucleotide differences), while the sequences of *P. necessarius* strain Ammermann and STIR1 differed only in a single base (99.98 % similarity). This single nucleotide polymorphism represents a synonymous substitution (T/C) at a third codon position of the gene (*icdA*) encoding the isocitrate dehydrogenase. The very high sequence similarity revealed at the eight loci is quite surprising since the sites of isolation of the two ciliates are located about 400 km apart in France (Marseille) and Italy (River Stirone near Parma), and in addition, the establishment of the Ammermann culture took place about 30 years before the isolation of *Euplotes* *aediculatus* STIR1 (Petroni* et al.*, 2002). This very high sequence similarity suggests, however, that the two strains also share very high genome-wide sequence similarities. Thus, the genome sequence of STIR1 could be considered as a surrogate for the unavailable genomic DNA of *P. necessarius* ‘stock 15’ (ATCC 30859).

**Fig. 2.**
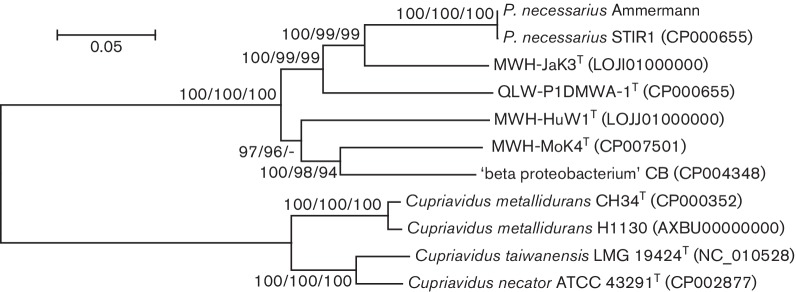
Neighbour-joining tree calculated with concatenated multilocus sequences of eight loci representing housekeeping genes of bacteria of the genus *Polynucleobacter* (Table S1). Sequences of the endosymbiont *P. necessarius* strain Ammermann were obtained by using specific primers, whereas all other sequences were extracted from whole genome sequences. Strain ‘beta proteobacterium’ CB represents a strain affiliated with the genus *Polynucleobacter* whose genome has been sequenced previously (Hao* et al.*, 2013).

**Fig. 3.**
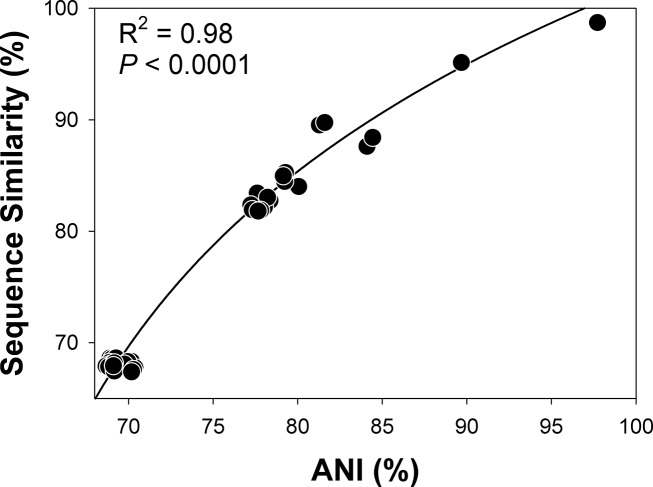
Correlation between ANI values obtained from whole genome comparisons and sequence similarities of the concatenated multilocus sequences (concatenated protein-coding loci listed in Table S1). The analysis included all taxa shown in Fig. 2 except *P. necessarius* strain Ammermann. The curve shown resulted from regression analysis with a three-parameter logarithmic equation. Note that the two data points with highest ANI and sequence similarity values resulted from comparisons of two strains representing the genus *Cupriavidus*, respectively.

Next, we analysed whether sequence similarities of multilocus sequences correlate with whole-genome ANI values. Concatenated sequences homologous to the eight loci and genome sequences of six strains representing the genus *Polynucleobacter* (including ‘beta proteobacterium’ CB) and four strains representing the genus *Cupriavidus* (Fig. 2) were analysed (Fig. 3). This revealed a tight correlation of sequence similarity of the eight concatenated loci and ANI values (R^2^ 0.98, *P*>0.0001), which enabled predictions of pairwise ANI values for the genomes of the four free-living strains investigated and *P. necessarius* strain Ammermann. The predicted values fell in the range of 77–85 % ANI, which confirmed that none of the four free-living strains should be classified as a member of the species *P. necessarius*. Since the multilocus sequences of *P. necessarius* strain Ammermann and STIR1 were almost identical, it can be assumed that even the whole genomes are quite similar. This justifies an alternative opportunity for the estimation of the genome similarities between the four free-living strains, respectively, and *P. necessarius* strain Ammermann by direct comparison with the STIR1 genome (Table 3). As expected, these comparisons also resulted in ANI values below 85 %.

Phylogenetic analyses of the concatenated multilocus sequences of bacteria of the genus *Polynucleobacter* and close relatives affiliated with the genus *Cupriavidus* resulted in separate clustering of members of these two genera (Fig. 2). Interestingly, the phylogenetic distances between taxa of the genus *Polynucleobacter* were quite large compared with distances obtained for strains representing distinct species of the genus *Cupriavidus*. The phylogenetic analyses performed further supports the separation of the four free-living strains representing the genus *Polynucleobacter* into four novel species.

Altogether, the ANI values obtained and the phylogenetic analysis of protein-encoding sequences enforce a revision of the current taxonomy of the species *P. necessarius*. All four free-living strains should be excluded from the species *P. necessarius* and transferred to novel species, respectively.

We propose to establish four novel species, *Polynucleobacter asymbioticus* comb. nov., *Polynucleobacter duraquae* sp. nov., *Polynucleobacter yangtzensis* sp. nov. and *Polynucleobacter sinensis* sp. nov., represented by the type strains QLW-P1DMWA-1^T^, MWH-MoK4^T^, MWH-JaK3^T^ and MWH-HuW1^T^, respectively. These proposed type strains were described previously (Hahn* et al.*, 2009), and data previously lacking for strain MWH-MoK4^T^ are presented in Table 4.

**Table 4.** Major fatty acid contents of strains representing the four novel species of the genus *Polynucleobacter* Data for strains QLW-P1DMWA-1^T^, MWH-JaK3^T^ and MWH-HuW1^T^ were taken from Hahn* et al.* (2009); however, fatty acid methyl esters had been prepared and measured under the same conditions as for strain MWH-MoK4^T^.

Fatty acid	QLW-P1DMWA-1^T ^	MWH-JaK3^T^	MWH-HuW1^T^	MWH-MoK4^T^
C_12 : 0_	3.4	3.7	5.5	3.8
C_14 : 0_	0.9	1.2	0.3	0.3
C_15 : 0_	0.3	–	0.3	–
C_16 : 0_	22.2	15.5	29.6	15.9
C_17 : 0_	–	–	0.5	–
C_18 : 0_	1.2	0.5	2.4	0.5
C_20 : 0_	1.1	–	–	–
C_14 : 1_ω5*c*	–	0.6	0.2	–
C_15 : 1_ω6*c*	–	–	0.6	–
C_16 : 1_ω5*c*	0.9	0.4	–	0.4
C_16 : 1_ω7*c*	41.3	35.6	45.0	38.6
C_18 : 1_ω9*c*	–	–	0.4	0.3
C_18 : 1_ω7*c*	12.9	20.4	1.1	19.8
11-Methyl C_18 : 1_ω7*c*	3.1	8.1	1.1	4.2
C_12 : 0_ 2-OH	2.5	2.2	1.3	1.3
C_16 : 0_ 2-OH	–	–	–	1.8
Summed feature 1 (including C_12 : 0_ ALDE?)	0.4	0.5	1.0	–
Summed feature 2 (including C_14 : 0_ 3-OH)	9.6	9.2	9.9	11.9
Summed feature 7 (including C_19__ : 1_ω6*c*)	0.4	2.0	0.3	–

*P. necessarius*, the four novel species and several undescribed taxa (Hahn *et al.*, 2016) together form the so-called species complex PnecC within the genus *Polynucleobacter* (Hahn, 2003; Jezbera *et al.*, 2011). This cryptic species complex is characterized by the presence of a diagnostic sequence (5′-GAGCCGGTGTTTCTTCCC-3′, *Escherichia coli* positions 445–463) in the 16S rRNA gene. This diagnostic sequence can be detected by using the PnecC-specific fluoresence *in situ* hybridization (FISH) probe PnecC-16S-445 (Hahn *et al.*, 2005). However, assignment to a certain species within this cryptic species complex cannot be based solely on ribosomal sequences.

Characteristics for differentiation of strains affiliated with the genus *Polynucleobacter* from other members of the family *Burkholderiaceae* were published previously (Hahn* et al.*, 2009). Differentiation of the four novel species of the genus *Polynucleobacter* from the previously described species *Polynucleobacter cosmopolitanus*,*Polynucleobacter acidiphobus*,*Polynucleobacter difficilis* and *Polynucleobacter rarus* is possible by using chemotaxonomic criteria. All four strains differ from strains representing *P. rarus*,*P. difficilis* and *P. acidiphobus* in the G+C content of DNA (Hahn* et al.*, 2011a, b, 2012a) but cannot be discriminated by this feature from strains of *P. cosmopolitanus* (Hahn* et al.*, 2010). Strains of the latter species are characterized by the presence of the fatty acid C_12 : 0_ 3-OH, which was not detected in any other species of the genus *Polynucleobacter* characterized so far.

The discrimination of the four novel species from each other is possible by using the criteria presented in Table 5. The type strain of *P. asymbioticus* comb. nov. is the only strain able to assimilate l-aspartate, while the type strain of *P. duraquae* sp. nov. is the only strain which showed no assimilation of propionate. The type strain of *P. sinensis* sp. nov. is the sole strain able to assimilate both oxaloacetate and l-glutamate, while *P. yangtzensis* sp. nov. is the only strain able to assimilate propionate but not both l-glutamate and L-aspartate. Furthermore, the type strain of *P. asymbioticus* comb. nov. differs from the three other type strains by the detection of the saturated fatty acid C_20 : 0_, while the type strain of *P. duraquae* sp. nov. differs from the others by the detection of the hydroxylated fatty acid C_16 : 0_ 2-OH (Table 4). The type strain of *P. sinensis* sp. nov. differs from the other three type strains by the detection of the two fatty acids C_17 : 0_ and C_15 : 1_ω6*c*; however, this trait may not be very reliable since both fatty acids contributed less than 1 % to the total fatty acids.

**Table 5.** Characteristics for differentiation of the four proposed novel species of the genus *Polynucleobacter* The method used for determination of assimilation capabilities was described previously (Hahn *et al.*, 2009).

Characteristic	QLW-P1DMWA-1^T ^	MWH-JaK3^T^	MWH-HuW1^T^	MWH-MoK4^T^
Assimilation of:				
Propionic acid	+	+	+	−
Oxaloacetic acid	−	+	+	+
l-Glutamate	+	−	+	−
l-Aspartate	+	−	−	−

## Emended description of *Polynucleobacter necessarius* Heckmann & Schmidt 1987 emend. Hahn *et al.* 2009

*Polynucleobacter necessarius*(Po.ly.nuc′le.o.bac.ter. Gr. adj. *polys* numerous; L. masc. n. *nucleous* nut, kernel; N.L. masc. n. *bacter* the equivalent of the Gr. neut. n. *bactron* a rod; N.L. masc. *Polynucleobacter* rod with many nucleoids; nec.es.sar′i.us. L. adj. *necessarius* indispensable, necessary).

This species belongs to the family *Burkholderiaceae* and harbours obligatory endosymbiotic strains living in ciliates of the genus *Euplotes.* So far, endosymbiotic strains could not be cultured in pure culture (Vannini *et al.*, 2007; Hahn *et al.*, 2009). Cells have elongated morphology with multiple nucleoid-like structures; penicillin-sensitive (Heckmann & Schmidt, 1987). Most probably descended from free-living strains of the genus *Polynucleobacter* (Boscaro* et al.*, 2013; Hahn* et al.*, 2009; Vannini* et al.*, 2007). Genome size is about 1.6 Mbp and its DNA G+C content is 44–46 mol%. The genome includes a large number of pseudogenes (Boscaro *et al.*, 2013; Vannini *et al.*, 2007), and the 16S rRNA gene sequence contains some unusual mutations not found so far in free-living strains of the genus *Polynucleobacter* (Vannini* et al.*, 2007). The type of the species *P. necessarius* is represented by a description of the endosymbionts in the currently unavailable culture ATCC 30859. Endosymbionts of the genus *Polynucleobacter* contained in the *Euplotes aediculatus* Ammermann culture (Muséum National d’Histoire Naturelle, Paris, France) are considered to be identical with the endosymbionts in culture ATCC 30859. Endosymbionts of the genus *Polynucleobacter* in the *Euplotes aediculatus* STIR1 culture were found to be highly similar genetically to the endosymbionts in the Ammermann culture. Therefore, this endosymbiont, characterized by a complete genome sequence, is also considered to be a member of the species *P. necessarius*. Gene and genome sequences characterizing these endosymbionts are available under the accession numbers AM397067, AM398078, LN998990-LN998998 and CP001010.

## Description of *Polynucleobacter asymbioticus* comb. nov.

*Polynucleobacter asymbioticus* (a.sym.bi.o′ti.cus. Gr. pref *a* not; N.L. masc. adj. *symbioticus -a -um* living together; N.L. masc. adj. *asymbioticus* not symbiotic).

Basonym: *Polynucleobacter necessarius *subsp. *asymbioticus* Hahn *et al.* 2009.

The description is based on phenotypical data of Hahn *et al.* (2009) and Hahn *et al.* (2012a), on chemotaxonomical data of Hahn *et al.* (2009), and on genomic data of Meincke *et al.* (2012), as well as data presented in Tables 1 and 2. Contains free-living strains of the genus *Polynucleobacter* dwelling in the water column of acidic or circum-neutral freshwater habitats. Cells are short rods, 0.7–1.2 µm in length and 0.4–0.5 µm in width. Chemo-organotrophic, aerobic and facultatively anaerobic. Can be cultivated on NSY, R2A, Luria–Bertani and peptone media. Colonies grown on NSY agar are non-pigmented, circular and convex with smooth surface. Growth occurs at up to 34 °C. Growth occurs with 0–0.4 % (w/v) NaCl. Weak growth occurs with 0.5 % (w/v) but not with 0.6 % (w/v) NaCl or higher concentrations. Assimilates acetate, propionate, pyruvate, malate, succinate, fumarate, d-galacturonic acid, l-cysteine, l-glutamate and l-aspartate. Weak assimilation is observed for some more substrates (Hahn* et al.*, 2009). Does not assimilate glycolate, oxalate, oxaloacetate, l-serine or citrate. Major cellular fatty acids are C_16 : 1_ω7*c*, C_16 : 0_, C_18 : 1_ω7*c* and summed feature 2 (including C_14 : 0_ 3-OH). The sole 2-hydroxylated compound is C_12 : 0_ 2-OH.

The type strain is QLW-P1DMWA-1^T^ (=DSM 18221^T^=CIP 109841^T^), which was isolated from a small acidic freshwater pond located in the Austrian Alps at an altitude of 1300 m (Hahn* et al.*, 2005). The genome of the type strain is characterized by a size of 2.2 Mbp and a DNA G+C content of 44.8 mol%. Strains affiliated with this species are characterized by the diagnostic sequence 5′-ACTAAGCGATCTAATGATTGTTTA-3′ in the 16S–23S rRNA (Jezbera* et al.*, 2011). Gene and genome sequences characterizing the type strain are available under the accession numbers CP000655 and AJ879783.

## Description of *Polynucleobacter duraquae* sp. nov.

*Polynucleobacter duraquae* (dur.a′quae. L. adj. *durus* -a -um hard; L. fem. n. *aqua* water; N.L. gen. fem. n. *duraquae* from/of hard water, i.e. water with higher concentrations of dissolved limestone).

The description is based on phenotypical data of Hahn (2003) and Hahn *et al.* (2009), on chemotaxonomical data of Hahn *et al.* (2009), data presented in Table 4 and genomic data presented in Table 1. Contains free-living strains of the genus *Polynucleobacter* dwelling in the water column of alkaline or circum-neutral freshwater systems. Never found in acidic waters (Hahn *et al.*, 2016; Jezbera* et al.*, 2011). Cells are curved rods, 0.9–2.9 µm in length and 0.4–0.5 µm in width. Chemo-organotrophic and aerobic; anaerobic growth was not observed. The type strain encodes a gene cluster for anoxygenic photosynthesis but expression of a photosynthesis system has not been observed so far. Encodes genes for synthesis of flagella but motility is usually not observed. Can be cultivated on NSY, peptone, yeast extract, R2A and Luria–Bertani media. Colonies grown on NSY agar are non-pigmented, circular and convex with smooth surface. Growth occurs at up to 30 °C. Growth occurs with 0–0.3 % (w/v) NaCl. Assimilates acetate, pyruvate, oxaloacetate, succinate, fumarate and l-cysteine. Weak assimilation is observed for some more substrates (Hahn* et al.*, 2009). Does not assimilate glycolate, glyoxylate, propionate, malonate, oxalate, levulinate, d-mannose, d-galactose, l-fucose, d-sorbitol, l-glutamate, l-aspartate, l-alanine, l-serine, l-asparagine or citrate. Major cellular fatty acids are C_16 : 1_ ω7c, C_18 : 1_ ω7c, C_16 : 0_ and summed feature 2 (including C_14 : 0_ 3-OH).

The type strain is MWH-MoK4^T^ (=DSM 21495^T^= CIP 110977^T^), which was isolated from alkaline Lake Mondsee (Hahn, 2003). The genome of the type strain is characterized by a size of 2.0 Mbp and a DNA G+C content of 45.2 mol%. Gene and genome sequences characterizing the type strain are available under the accession numbers CP007501 and AJ550654.

## Description of *Polynucleobacter yangtzensis* sp. nov.

*Polynucleobacter yangtzensis* (yang.tzen′sis. N.L. masc. adj. *yangtzensis* of or belonging to the Yangtze River, the river from where the type strain was isolated).

The description is based on phenotypical data of Hahn (2003) and Hahn *et al.* (2009), on chemotaxonomical data of Hahn *et al.* (2009) and on genomic data presented in this study (Table 2). Contains free-living strains of the genus *Polynucleobacter* dwelling in the water column of freshwater systems. Cells are short rods, 0.5–1.5 µm in length and 0.3–0.5 µm in width. Chemo-organotrophic, aerobic and facultatively anaerobic. Can be cultivated on NSY, nutrient broth, peptone, soytone, yeast extract, tryptic soy media, Standard agar and R2A media. Colonies grown on NSY agar are non-pigmented, circular and convex with smooth surface. Growth occurs at up to 35 °C. Growth occurs with 0–0.3 % (w/v) NaCl. Assimilates acetate, propionate, pyruvate, oxaloacetate, malate, succinate, fumarate and l-cysteine. Weak assimilation is observed for some more substrates (Hahn* et al.*, 2009). Does not assimilate glycolate, glyoxylate, oxalate, levulinate, d-mannose, d-glucose, d-galactose, d-sorbitol, l-glutamate, l-aspartate, l-alanine, l-serine, l-asparagine or citrate. Major cellular fatty acids are C_16 : 1_ω7*c*, C_18 : 1_ω7*c*, C_16 : 0_ and summed feature 2 (including C_14 : 0_ 3-OH and iso-C_16 : 0_ I). The sole 2-hydroxylated compound is C_12 : 0_ 2-OH.

The type strain is MWH-JaK3^T^ (=DSM 21493^T^=CIP 110976^T^), which was isolated from the Yangtze River (Hahn, 2003). The species epithet does not indicate that the distribution of the taxon is restricted to a certain geographic area. The genome of the type strain is characterized by a size of 2.0 Mbp and a DNA G+C content of 45.4 mol%. Gene and genome sequences characterizing the type strain are available under the accession numbers LOJI00000000 (version LOJI01000000) and AJ550657.

## Description of *Polynucleobacter sinensis* sp. nov.

*Polynucleobacter sinensis* (sin.en′sis. N.L. fem. adj. *sinensis* pertaining to China, the country where the bacterium was isolated).

The description is based on phenotypical data of Hahn *et al.* (2009), on chemotaxonomical data of Hahn *et al.* (2009) and on genomic data presented in this study (Table 2). Contains free-living strains of the genus *Polynucleobacter* dwelling in the water column of freshwater systems. Cells are curved rods, 0.6–1.4 µm in length and 0.4–0.5 µm in width. Chemo-organotrophic and aerobic; anaerobic growth is not observed. Shows good growth on NSY and R2A media. Colonies grown on NSY agar are non-pigmented, circular and convex with smooth surface. Growth occurs at up to 35 °C. Growth occurs with 0–0.5 % (w/v) NaCl. Assimilates acetate, propionate, malonate, malate, pyruvate, oxaloacetate, succinate, fumarate and l-glutamate. Weak assimilation is observed for l-cysteine. Does not assimilate glycolate, glyoxylate, oxalate, levulinate, d-mannose, d-glucose, d-galactose, d-lyxose, d-fructose, l-fucose, d-sorbitol, l-aspartate, l-alanine, l-serine, l-asparagine or citrate. Major cellular fatty acids are C_16 : 1_ω7*c*, C_16 : 0_ and summed feature 2 (including C_14 : 0_ 3-OH and C_16 : 0_-iso I). The sole 2-hydroxylated compound is C_12 : 0_ 2-OH.

The type strain is MWH-HuW1^T^ (=DSM 21492^T^=CIP 110978^T^), which was isolated from a slightly alkaline, artificial pond (31° 20′ 14.81″ N 120° 34′ 34.20″ E) at Tiger Hill (Huqiu) located in Suzhou, PR China (Hahn, 2003). The species epithet does not indicate that the distribution of the taxon is restricted to a certain geographic area. The genome of the type strain is characterized by a size of 2.3 Mbp and a DNA G+C content of 45.5 mol%. Gene and genome sequences characterizing the type strain are available under the accession numbers LOJJ00000000 (version LOJJ01000000) and AJ550666.

## Supplementary Data

255 Supplementary File 1
