# Supplementary material for: Reclassification of four Polynucleobacter necessarius strains as representatives of Polynucleobacter asymbioticus comb. nov., Polynucleobacter duraquae sp. nov., Polynucleobacter yangtzensis sp. nov. and Polynucleobacter sinensis sp. nov., and emended description of Polynucleobacter necessarius
Source: Int J Syst Evol Microbiol. 2016 Aug;66(8):2883–92. doi: 10.1099/ijsem.0.001073 (PMC5018217; doi:10.1099/ijsem.0.001073)
Supplement: Supplementary file 1 [file jgv-66-2883-s001.pdf]

Hahn, M.W., J. Schmidt, A. Pitt, S.J. Taipale, and E. Lang. **Reclassification of four *Polynucleobacter necessarius* strains as *Polynucleobacter asymbioticus* comb. nov., *Polynucleobacter duraquae* sp. nov., *Polynucleobacter yangtzensis* sp. nov., and *Polynucleobacter sinensis* sp. nov., and emended description of the species *Polynucleobacter necessarius***. Int. J. Syst. Evol. Microbiol.

Supplementary Materials Table S1. PCR primers used for amplification and sequencing of loci of the *P. necessarius* Ammermann genome.

Locus	Product	Position	Forward Primer		Reverse Primer	
16S-23S ITS		35480	27F[§] (Lane, 1991)		STIR1-23sR-R1	5'-GTCGCCTGTAATCGCC-3'
rpoB	DNA polymerase III beta subunit	45559	STIR-rpoB-F	5'-TACTGATGAAACAGCAGACC-3'	STIR-rpoB-R	5'-TTATCAATCGTTGCATTCGCC-3'
trpE	Anthranilate synthase	142042	STIR-trpE-F	5'-CTTTACGTTAAGCTCAGCC-3'	STIR-trpE-R	5'-CCAAGCACTCATCTTCTTC-3'
icdA	Isocitrate dehydrogenase [NADP]	332904	STIR-icdA-F	5'-ATTCGTACGCGCTACTCC-3'	STIR-icdA-R	5'-CAAAATTGCCGTGTGTCTTAC-3'
glnA	Glutamine Synthase	607079	glnA1212F (Jezbera *et al.*, 2011)		glnA1895R (Jezbera *et al.*, 2011)	
mdh	Malate dehydrogenase	926803	STIR-mdh-F	5'-ATGCGTGTCGCCGTAAC-3'	STIR-mdh-R	5'-TTAACGCCAGCTTGTTCTTC-3'
fbp	Fructose-1,6-bisphosphatase	1026731	STIR-fbp-F	5'-CTTACCAACCGGCCTGC-3'	STIR-fbp-R	5'-CCATTAGTAGAGACGCCAC-3'
msbA	Lipid A export permease protein	1077469	STIR-msbA-F	5'-GCCTTACCGGTCAGCTC-3'	STIR-msbA-R	5'-TTCCGCCTTTGGTCTTGTC-3'
gyrA	DNA gyrase subunit A	1139469	STIR-gyrA-F	5'-CGTGCCAAGACTCACTTC-3'	STIR-gyrA-R	5'-ACGTTCTGGCTTAGCTAAC-3'

[§] Primer Poly1406f (5'-CTT GTA CAC ACC GCC CGT-3') was used for sequencing

References

Jezbera, J., Jezberova, J., Brandt, U. & Hahn, M. W. (2011). Ubiquity of *Polynucleobacter necessarius* subspecies *asymbioticus* results from ecological diversification. Environmental Microbiology 13, 922-931.

Lane, D. J. (1991). 16S/23S rRNA sequencing. In Nucleic acid techniques in bacterial systematics, pp. 115-175. Edited by E. Stackebrandt & M. Goodfellow. New York: John Wiley and Sons.
